# Bistability and Oscillations in the Huang-Ferrell Model of MAPK Signaling

**DOI:** 10.1371/journal.pcbi.0030184

**Published:** 2007-09-28

**Authors:** Liang Qiao, Robert B Nachbar, Ioannis G Kevrekidis, Stanislav Y Shvartsman

**Affiliations:** 1 Department of Chemical Engineering, Princeton University, Princeton, New Jersey, United States of America; 2 Applied Computer Science and Mathematics, Merck Research Laboratories, Rahway, New Jersey, United States of America; 3 Program in Applied and Computational Mathematics, Princeton University, Princeton, New Jersey, United States of America; 4 Lewis-Sigler Institute for Integrative Genomics, Princeton, New Jersey, United States of America; California Institute of Technology, United States of America

## Abstract

Physicochemical models of signaling pathways are characterized by high levels of structural and parametric uncertainty, reflecting both incomplete knowledge about signal transduction and the intrinsic variability of cellular processes. As a result, these models try to predict the dynamics of systems with tens or even hundreds of free parameters. At this level of uncertainty, model analysis should emphasize statistics of systems-level properties, rather than the detailed structure of solutions or boundaries separating different dynamic regimes. Based on the combination of random parameter search and continuation algorithms, we developed a methodology for the statistical analysis of mechanistic signaling models. In applying it to the well-studied MAPK cascade model, we discovered a large region of oscillations and explained their emergence from single-stage bistability. The surprising abundance of strongly nonlinear (oscillatory and bistable) input/output maps revealed by our analysis may be one of the reasons why the MAPK cascade in vivo is embedded in more complex regulatory structures. We argue that this type of analysis should accompany nonlinear multiparameter studies of stationary as well as transient features in network dynamics.

## Author Summary

Molecular studies of cell communication systems lead to models with multiple free parameters. Analysis of dynamical behavior of these models presents considerable challenge. We have developed a computational approach for the efficient exploration of dynamic behavior in such models and applied this method to the model of the Mitogen Activated Protein Kinase cascade, a signaling network conserved in all eukaryotes. Previous analysis of this model suggested that it works as a reversible switch. We have shown that it can also function as an irreversible switch and as a clock.

## Introduction

Physicochemical models of signaling pathways are characterized by high levels of structural and parametric uncertainty [1–7], reflecting both incomplete knowledge about signal transduction and the intrinsic variability of cellular processes. As a result, these models try to predict the dynamics of systems with tens or even hundreds of free parameters [8–10]. At this level of uncertainty, model analysis should emphasize statistics of systems-level properties, rather than the detailed structure of solutions or boundaries separating different dynamic regimes [11–18]. Chemical network theory and monotone systems approaches can characterize dynamics of biochemical networks based only on their structure, independently of a particular choice of parameters [19–21]. Under certain conditions, these methods can rule out whole classes of behaviors, such as bistability or oscillations, but they do not provide information about the relative prevalence of coexisting dynamic patterns. At the other extreme of model analysis techniques are continuation algorithms, which track steady states or limit cycles as a function of just one or two model parameters at a time [9,22].

**Figure 1:**
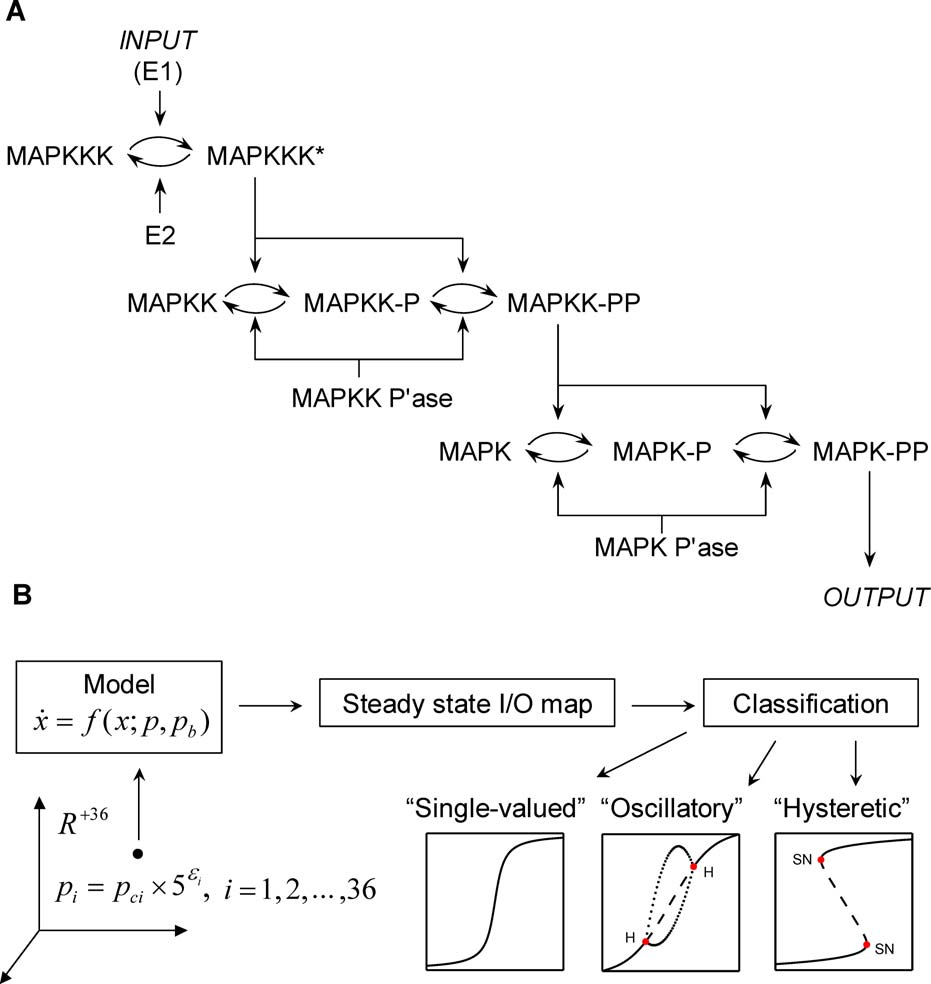
MAPK Cascade Structure and Schematic of the Statistical Parametric Analysis Procedure (A) Schematic of the MAPK cascade, reproduced from [26], 1996. The full MAPK cascade consists of ten enzymatic reactions where each step is modeled by mass action; it can be described by an ODE system consisting of 15 variables and 37 parameters. The distinguished (bifurcation) parameter, *p_b_* ∈ *R* ^+^, is the cascade input (the total concentration of E1). The 36-dimensional vector of the remaining model parameters (*p* ∈ *R* ^+36^) consists of 30 rate constants and six total concentrations). (B) Schematic of the sampling/continuation approach. The components of the remaining model parameters are generated from the 36-dimensional vector of predefined base values (*p_c_* ∈ *R* ^+36^) and the random variables *ɛ_i_* that are uniformly distributed in [−*q*, *q*], where *q* is the size of the uncertainty interval: *p_i_* = *p_ci_* × 5^ɛi^, *i* = 1, 2, … 36.

While the information provided by continuation methods is only local, they can be efficiently combined with random parameter sampling algorithms, enabling the statistical exploration of systems-level properties, such as stability and robustness [23,24]. Here, we use this approach to characterize the statistics of steady-state input/output maps in the model of the Mitogen Activated Protein Kinase (MAPK) cascade, a network present in all eukaryotic cells and one of the most extensively modeled signaling systems [25]. The first model of the MAPK cascade was developed by Huang and Ferrell, and used as a basis for connecting the structure of the cascade and its dynamics (Figure 1A). Based on mass-action kinetics, the model described the dynamics of 22 species participating in ten reactions [26]. Each of the 37 model parameters, which have been either estimated or extracted from cellular and biochemical experiments, was specified within a reasonably broad interval. Huang and Ferrell hypothesized that the three-tiered structure of the MAPK cascade controls its steady-state input–output behavior. Based on simulations with hundreds of randomly generated parameter sets, they found that the input–output map is ultrasensitive. Importantly, this prediction was supported by biochemical experiments in Xenopus oocyte extracts [26].

In a later sequence of papers, Ferrell and co-workers demonstrated that ultrasensitivity can lead to bistability in positive feedback networks, in which the activated MAPK positively regulates the input to the cascade [27–29]. Recently, however, Kholodenko and co-workers have established that bistability is possible at the level of a single stage of the MAPK cascade [30]. Specifically, when the same phosphatase (e.g., MAPK'Pase) dephosphorylates both the monophosphorylated and double-phosphorylated forms of the substrate (e.g., MAPK), the double-phosphorylated form competitively inhibits the second dephosphorylation. In combination with the conservation of the total amount of substrate, this generates an equivalent of a direct positive feedback and can lead to bistability [30,31]. The extent to which this single-stage phenomenon influences the dynamics of the entire MAPK cascade has been unclear. Here, we demonstrate that a significant fraction of the multidimensional parameter space in the Huang-Ferrell model exhibits bistability and oscillations. Furthermore, our computational results strongly suggest that single-stage bistability is a necessary condition for the oscillatory behavior at the cascade level.

## Results

### Computational Discovery of Bistable and Oscillatory Input–Output Maps

We used a combination of parameter sampling and continuation algorithms to characterize the statistics of input–output (I/O) maps in the Ferrell-Huang model [26]. Just as in the original publication, the I/O map describes the system response, taken to be the fraction of MAPK in the double-phosphorylated state, as a function of a distinguished model parameter, the input to the first stage of the cascade (Figure 1A). Specifically, the 36-dimensional vector of the remaining model parameters was repeatedly generated by Monte Carlo sampling from the hypercube defined by Huang and Ferrell (Table S1). For each of the generated parameter sets, a pseudoarclength-continuation algorithm was used to compute the steady-state I/O map [32]. This approach can both locate steady states and characterize their stability as a function of the input to the cascade. We developed a classification procedure for assigning the I/O maps to one of the three categories: “single-valued,” “oscillatory,” and “hysteretic” (Figure 1B; see Protocol S1 for details of the sampling, continuation, numerical stability analysis, and classification protocols).

**Figure 2:**
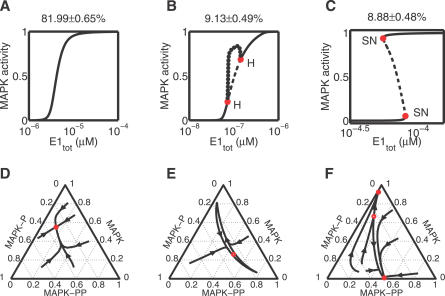
Classes of the Dynamics of the MAPK Cascade and Their Estimated Frequencies (A–C) Representative bifurcation diagrams and the corresponding frequencies of the three categories (from left to right, “single-valued,” “oscillatory,” and “hysteretic”). Hopf and Saddle-Node bifurcation points are marked in red and denoted by “H” and “SN”, respectively. (D–F) Representative phase diagrams corresponding to E1_tot_ = 10^−5.5^, 10^−7^, and 10^−4.1^ *μ*M as in (A–C), respectively.

The summary of the classification results, based on 20,000 parameter sets, is presented in Figure 2. We found that ∼80% of the generated models led to single-valued I/O maps (Figure 2A). Surprisingly, the rest of the generated models corresponded to strongly nonlinear I/O maps. Specifically, ∼10% of models had I/O maps with regions of oscillations (Figure 2B), while ∼10% of models were bistable (Figure 2C; see Table S2 for examples). While the existence of bistable I/O maps could have been expected on the basis of the single-stage results by Kholodenko et al., our results provide the first evidence of oscillatory behavior in the MAPK cascade in the absence of explicit negative feedback [30,33]. The large sample size in our calculations ensured tight confidence intervals for these estimates of the frequencies of the three different classes of I/O diagrams (see also Figure S2). All of the bistable I/O maps had their left-most turning point for positive values of the input. Thus, we did not observe bistability at zero values of the input; such diagrams were proposed to mediate irreversible cell-fate transitions in Xenopus oocyte maturation [29].

Based on the results of our sampling/continuation approach, we characterized the statistical properties of the I/O maps. By fitting the single-valued I/O maps to Hill functions, we found that, with high probability, they are ultrasensitive, i.e., are characterized by high Hill constants (*n_H_* > 1), Figure S1). In particular, with probability ∼74%, single-valued I/O map is characterized by a Hill coefficient greater than 2: *P*(*n_H_* > 2) ≈ 0.74. Focusing on the hysteretic and oscillatory maps, we established that they involve concentration ranges that can be adequately described by a deterministic approach, i.e., they are characterized by reasonably large molecular copy numbers for all of the model components (assuming the volume of an oocyte cell is ∼1 *μ*L, a concentration even as low as 10^−9^ *μ*M still corresponds to approximately 600 molecules). The oscillatory solutions in the model were of the relaxation type, their amplitudes spanned the entire dynamic range of the outputs (from unphosphorylated to fully phosphorylated MAPK, Figure S3E), and their periods were quite long (typically ½ hour, Figure S4). See Figure S3 for a summary of the statistical properties of oscillatory and bistable regimes.

**Figure 3:**
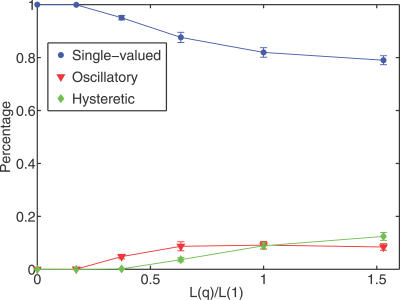
Frequencies of the Three I/O Map Classes Categories for the MAPK Cascade versus the Magnitude of the Uncertainty The function *L*(*q*) is defined as *L*(*q*) ≡ 5*^q^* − 5^−*q*^, where *q* is described in Figure 1B.

The upper and lower boundaries of the suggested range for each of the parameters in the original Huang-Ferrell paper were given by one-fifth and five times the mean parameter value, respectively [26]. Using our sampling/continuation approach, we found that oscillatory and bistable I/O maps occur for much smaller ranges of parametric uncertainty (Figure 3). Thus, the existence of deterministic oscillations and bistability is a robust property of the Huang-Ferrell model.

### Constructing and Deconstructing Oscillations in the MAPK Cascade

In the next set of computational studies, we explored the origin of oscillatory and bistable regimes. To simplify the notation, we label the different stages of the full MAPK cascade, i.e., the activation of MAPKKK, double-phosphorylation of MAPKK, and MAPK, with the numbers 1, 2, and 3, respectively, and use terms like “system 2”, “system 2+3” or “system 1+2+3” to indicate different reaction networks consisting of a single stage, two consecutive stages, or all stages of the full MAPK cascade, respectively. As a first step towards the analysis of the full model, we used our sampling/continuation approach to characterize the statistics of I/O maps in all possible single-stage and two-stage subsets of the full model (Table 1).

**Table 1:**
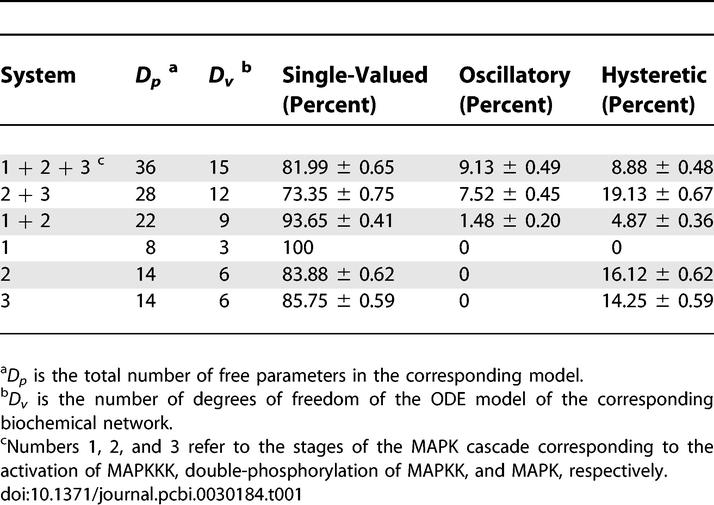
Frequencies of the I/O Map Classes for the Subsystems within the MAPK Cascade

As expected on the basis of previous analytical and computational results [30,34–36], we observed that the first stage is always monostable, while the second and third stages, each of which is formed by two consecutive double phosphorylation–dephosphorylation cycles, supports bistability. While bistability exists already at a single-stage level, our results strongly suggest that the emergence of oscillations requires at least two stages, one of which should be based on double phosphorylation (Table 1). Based on this, we hypothesized that the existence of single-stage bistability is a necessary condition for oscillations in multistage networks.

To test this hypothesis, we checked whether multistage networks with oscillatory I/O maps contain bistable single stages as their building blocks. Remarkably, for all possible multistage networks, i.e., system 1+2+3, 1+2, 2+3, we observed that oscillatory behavior requires at least one bistable single-stage module, e.g., stage 2 or 3 being bistable for the 1+2+3 system (Table 2). Note that there are no qualitative differences between the two-stage and three-stage cascade networks, with respect to their ability to support bistability and oscillations. Interestingly, this correlation between single-stage and multistage dynamics does not necessarily hold for bistable I/O maps (Table 2): multistage bistability can emerge from coupling of monostable stages.

**Table 2:**
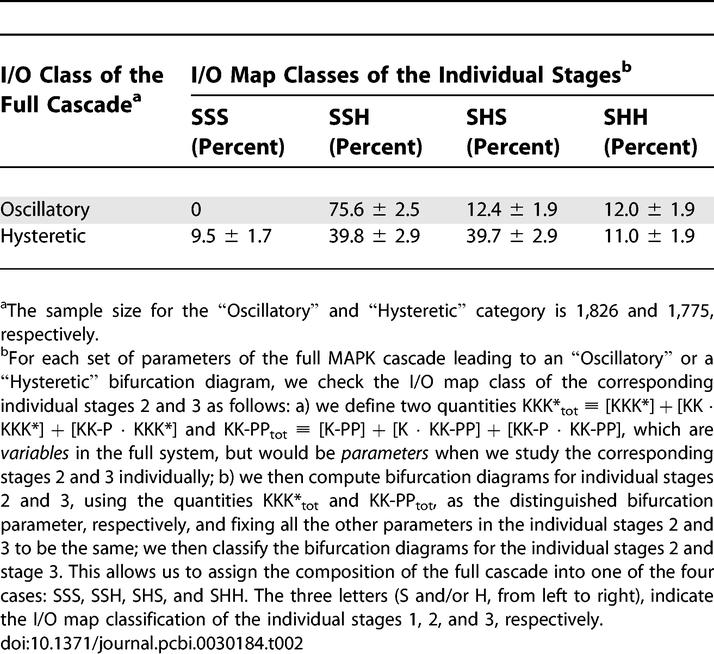
Analyzing the Nonlinear I/O Maps in the Full Cascade and Correlating the I/O Map in Relation to the I/O Map Classes of the Constituent Stages

**Figure 4:**
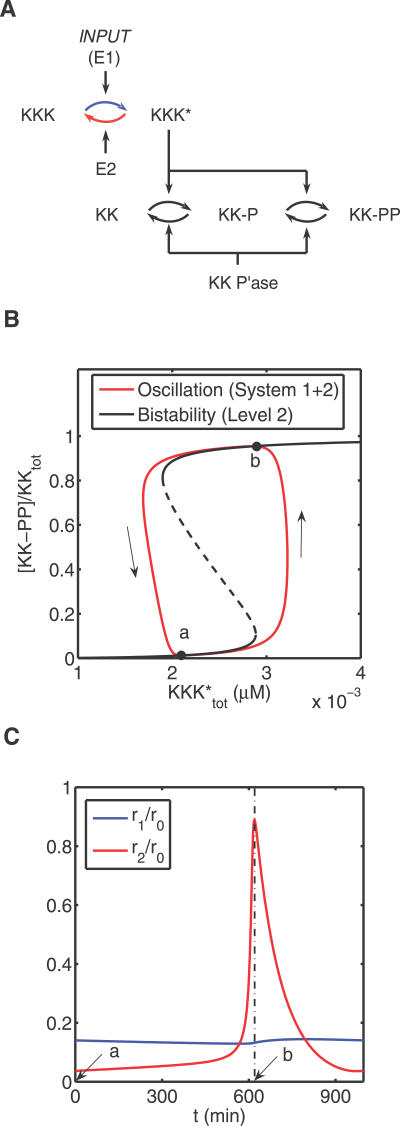
From Bistability to Oscillations (A) The structure of the “1+2” subsystem. MAPKKK activation and MAPKKK* deactivation are indicated by the blue and red arrows, respectively. (B) Limit cycle in the “1+2” system and its connection to the bistability in the “2” system. The limit cycle is plotted in red and the direction of time evolution is indicated by arrows. The hysteresis curve (black) is the I/O map of the system “2”, computed using the quantity KKK*_tot_ as a bifurcation parameter. KKK*_tot_, defined as KKK*_tot_ ≡ [KKK*] + [KK · KKK*] + [KK-P · KKK*], is a *variable* in system 1 + 2, but a *parameter* for system 2. (C) Dynamics of the rates of MAPKKK activation (r_1_) and MAPKKK* deactivation (r_2_) along the periodic trajectory. The rates have been normalized by the maximum MAPKKK activation (r_0_). The arrows correspond to different phases of the periodic trajectory in (B).

We subsequently analyzed the connection between multistage limit cycles and single-stage bistability. As expected from the established correlation between single-stage bistability and multistage oscillations, we found that, in all cases, multistage limit cycles are “built” around hysteresis loops of bistable single stages (Figure 4B shows an example of such a correlation). This might explain the predominantly relaxation character of the oscillations in the MAPK cascade (see above); this strongly suggests the relation between the modularity of the network structure and modularity of network dynamics.

By analyzing the rates of individual reactions along the limit cycle, we established that multistage oscillations rely on the backwards coupling between a bistable stage and the preceding stage in the cascade (e.g., Figure 4A). Specifically, when the bistable stage is in the “off” state (point “a” in Figure 4B), the kinase which carries out both of the phosphorylations within this stage is complexed with its substrates. As a consequence, it is protected from dephosphorylation by the phosphatase in the preceding stage, and the total concentration of the kinase gradually increases (r_1_ > r_2_ in Figure 4C). However, when the bistable stage switches to the “on” state (point “b” in Figure 4B), at a high total concentration of the kinase, this kinase runs out of substrates and itself becomes a substrate for the upstream phosphatase. As a result, the total concentration of this kinase decreases (r_1_ < r_2_ in Figure 4C). At low levels of kinase activity, the substrates of this kinase within the bistable stage quickly become dephosphorylated, and, eventually, the stage quickly undergoes the transition back to the “off” state. We have established that this simple sequence of events accounts for oscillations in all observed multistage systems within the MAPK cascade (Table 1). Thus, the oscillatory solutions, which were identified on the basis of a brute force computational approach, turned out to have a transparent mechanistic origin.

Finally, we assessed the possibility of synthesizing the multistage oscillations from individual components. For this, we estimated the probability that a single, randomly generated bistable stage would lead to oscillations when embedded within the MAPK cascade (Table 3). The results of this analysis strongly suggest that single-stage bistability is a necessary but not a sufficient condition for multistage oscillations. The same results also show that single-stage bistability is also not sufficient for generating the bistable multistage I/O maps. At the same time, the odds of observing cascade-level oscillations are greatly increased (more than 3-fold) by the presence of single-stage bistability (based on the data in Table 3).

**Table 3:**
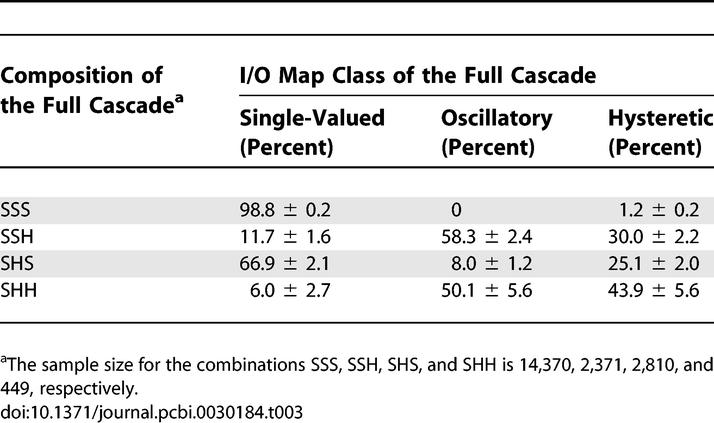
Frequencies of the I/O Map Classes of the Full Cascade versus the I/O Map Class of the Cascade Components

## Discussion

Based on the combination of random parameter search and continuation algorithms, we developed a methodology for the statistical analysis of mechanistic signaling models. In applying it to the well-studied MAPK cascade model, we discovered a large region of oscillations and explained their emergence from single-stage bistability. At this time, it is unclear whether such oscillations and bistability exist within the isolated MAPK cascade. However, our results suggest that oscillations and bistability do not necessarily imply the presence of explicit feedback loops.

The surprising abundance of strongly nonlinear (oscillatory and bistable) input/output maps revealed by our analysis may be one of the reasons why the MAPK cascade in vivo is embedded in more complex regulatory structures [9]. Numerous feedbacks targeting the MAPK circuit may either enhance the nonlinear behavior, e.g., by extending the range of inputs supporting bistability and oscillations, or eliminate it altogether, converting the switch-like behavior into a graded I/O response. In addition to feedbacks, synthesis and degradation of pathway components or their nucleocytoplasmic shuttling can affect the MAPK cascade dynamics [37–40]. The effects of these processes on the cascade dynamics can be systematically explored within our continuation/sampling approach.

Our objective has been to characterize the relative abundance of qualitatively different types of I/O maps. The rapid convergence of these estimates is an intrinsic feature of the Monte Carlo integration algorithms, which have been used in computational statistical physics for more than half a century. Hence, these kinds of approaches to statistical exploration of network dynamics will be effective whenever the outcomes of computations can be assigned to a finite number of classes. In our case, the outcomes of continuation runs were classified as “single-valued,” “oscillatory,” and “hysteretic” (see Protocol S1). In a different context, it may be important to characterize the statistics of transients induced by changes in the network inputs [41–43]. Given an appropriate classifier for transient solution features, one can identify the regions of the parameter space that lead to either adapting or sustained responses [40,42,44].

Recent single-cell measurements of protein levels show that they are characterized by high levels of variability. For example, measurements with GFP-labeled proteins in yeast and mammalian cells reported coefficients of variation around 20% [45,46]. Within this context, one can ask how robustly it is possible to guarantee a given type of network function. A computational approach to addressing this question can rely on the combination of a simple probability model for protein levels with a deterministic continuation algorithm. In this way, one can estimate the probability that a given I/O map will change its class, e.g., become oscillatory instead of hysteretic, when the model parameters are sampled from the multivariable distribution localized in parameter space.

**Figure 5:**
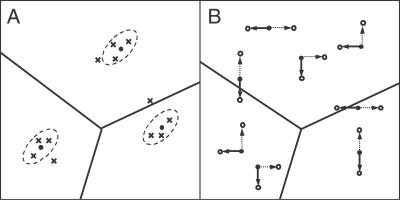
Caricature of the Robustness Tests for the I/O Maps (A) Regimes of different I/O maps are separated by solid lines. For each type of I/O map, a single parameter set (marked by a dot) is selected and used as the mean vector, around which multiple parameter sets (marked by crosses) are randomly sampled according to a multivariate normal distribution. I/O maps corresponding to the sampled parameter sets (marked by crosses) are then computed and categorized. (B) For each possible I/O map category, multiple parameter sets (marked by dots) are selected uniformly (in the log-space). For each of these parameter sets, the values of two randomly chosen parameters (which could be the same) are multiplied by ½ (for simulated gene deletion) and 2 (for simulated gene duplication), one for each, leading to two different parameter sets as indicated by the solid arrow and the dashed arrow, respectively. For each type of “mutation,” I/O maps for the sampled parameter sets (marked by circles) are computed and categorized.

Figure 5A presents an illustrative example of this type of calculation. Here we took the single-valued I/O map and perturbed it by sampling the parameters from the multivariable normal distribution, with means equal to the base values of parameters in the Huang-Ferrell model and coefficients of variation equal to 0.2. For this particular choice of the base model parameters and probability model, the I/O map remains single-valued (see Table 4), i.e., the classification of the I/O map as single-valued is robust. This is not, however, true in general, since in other regions of the parameter space one can easily find single-valued I/O maps that become either oscillatory or hysteretic upon localized variations of model parameters (unpublished data). Given the fact that these types of calculations are quite inexpensive at this time, we argue that this type of analysis should accompany multiparameter nonlinear studies of network dynamics.

**Table 4:**
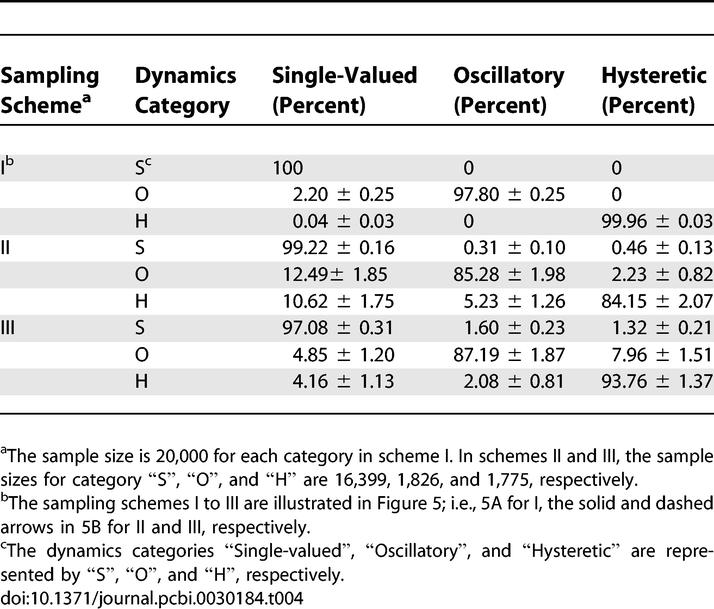
Transition Probabilities of the I/O Maps of the MAPK Cascade with Respect to Changes in the Protein Levels

Another motivation for a more detailed analysis of the distribution of different types of I/O maps in the multidimensional parameter space is provided by problems related to the evolutionary dynamics of signaling networks [47,48]. Mutations in the genes which encode components of signaling networks can affect both the protein levels and the rate-constants for protein/protein interactions. One can think that mutations in the regulatory sequence may translate into protein abundance, while mutations in the coding sequence may affect the protein activity and, hence, the rate constants in the model [43]. Depending on their location within the gene sequence, these changes can lead to either small or large shifts in the space of model parameters. Given a model of a mutational process and a biochemical and biophysical understanding of the connection between the gene sequence and protein abundance, one can systematically explore the connection between the dynamics of the genotype and network dynamics. For example, one can ask how easily a given mutational process can lead to a qualitative change of the I/O map. As an example, we computed the class change probabilities of the three different I/O maps in the Huang-Ferrell model upon simulated gene deletions and duplications (Figure 5B, Table 4). A similar type of approach may prove useful for interpreting the population level data on sequence variations in genes within the MAPK and other signaling pathways [49].

## Materials and Methods

The mathematical model of the MAPK cascade, described in Text S1, can be reduced to an equivalent Ordinary Differential Equation (ODE) system (Text S2). The procedure of Monte-Carlo sampling, pseudoarclength continuation, and categorization of the steady-state I/O maps for the reduced ODE system is described in Protocol S1. Numerical integration, used in obtaining initial guesses for steady states and for approximating oscillatory solutions, was performed using the stiff solver ODE15S in MATLAB, a commercial software package available at http://www.mathworks.com/. Numerical computations of steady-state solutions and stability/bifurcation analysis were performed in MATLAB code. The statistical frequencies in Figures 2 and 3 and Tables 1–4 are reported with 95% confidence intervals.

## Supporting Information

Figure S1 Cumulative Distribution Function of Hill Coefficients for “Single-Valued” I/O Maps (58 KB PDF)

Figure S2 Examples of More Complex Bifurcation Diagrams (67 KB PDF)

Figure S3 Various Statistics of I/O Maps (79 KB PDF)

Figure S4 Distribution of the Periods of Oscillatory Solutions for the MAPK Cascade and a Representative Limit Cycle (53 KB PDF)

Table S1 The Range of Sampled Model Parameters (51 KB PDF)

Table S2 Representative Parameter Sets for Oscillations and Hysteresis (52 KB PDF)

Text S1 Reactions within the MAPK Cascade and the Corresponding Differential Algebraic Equations (DAEs) (55 KB PDF)

Text S2 Reduction of the DAEs to an ODE System and Stability of the Corresponding Steady States (74 KB PDF)

Protocol S1 Sampling, Computation, and Classification of the Steady-State I/O Maps of the MAPK Cascade (65 KB PDF)

## Abbreviations

I/O: input–output
MAPK: Mitogen Activated Protein Kinase
ODE: ordinary differential equation
