# Supplementary material for: Bistability and Oscillations in the Huang-Ferrell Model of MAPK Signaling
Source: PLoS Comput Biol. 2007 Sep 28;3(9):e184. doi: 10.1371/journal.pcbi.0030184 (PMC1994985; doi:10.1371/journal.pcbi.0030184)
Supplement: Figure S1 — (58 KB PDF) [file pcbi.0030184.sg001.pdf]

Figure S1

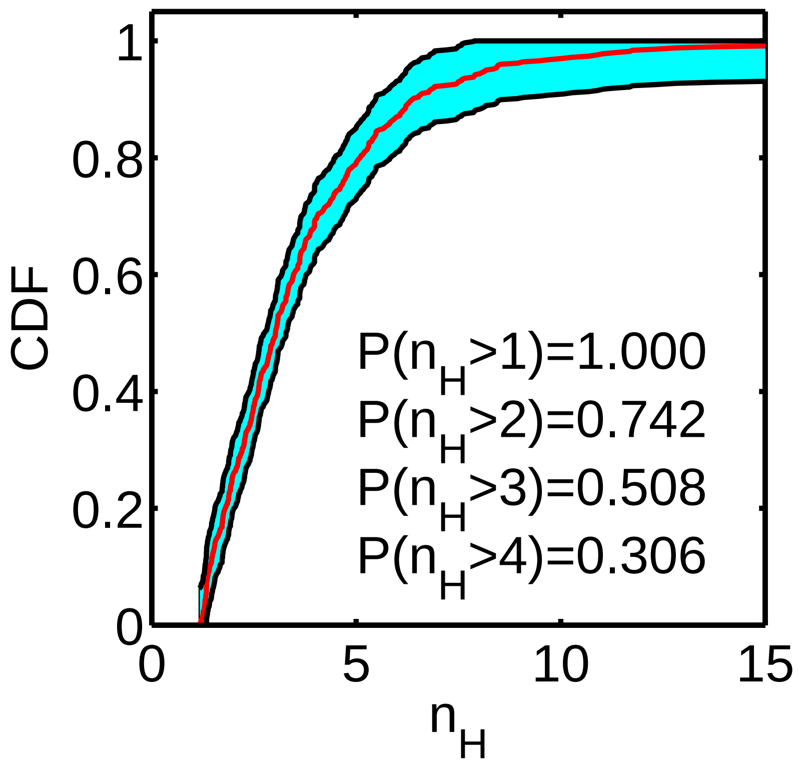

The Cumulative Distribution Function (CDF) of the Hill coefficients (n_H) for "Single-valued" steady state I/O maps (red solid line) is plotted with a 95% confidence band (in cyan) for a sample size $n = 500$.
