# Supplementary material for: Bistability and Oscillations in the Huang-Ferrell Model of MAPK Signaling
Source: PLoS Comput Biol. 2007 Sep 28;3(9):e184. doi: 10.1371/journal.pcbi.0030184 (PMC1994985; doi:10.1371/journal.pcbi.0030184)
Supplement: Figure S2 — (67 KB PDF) [file pcbi.0030184.sg002.pdf]

Figure S2

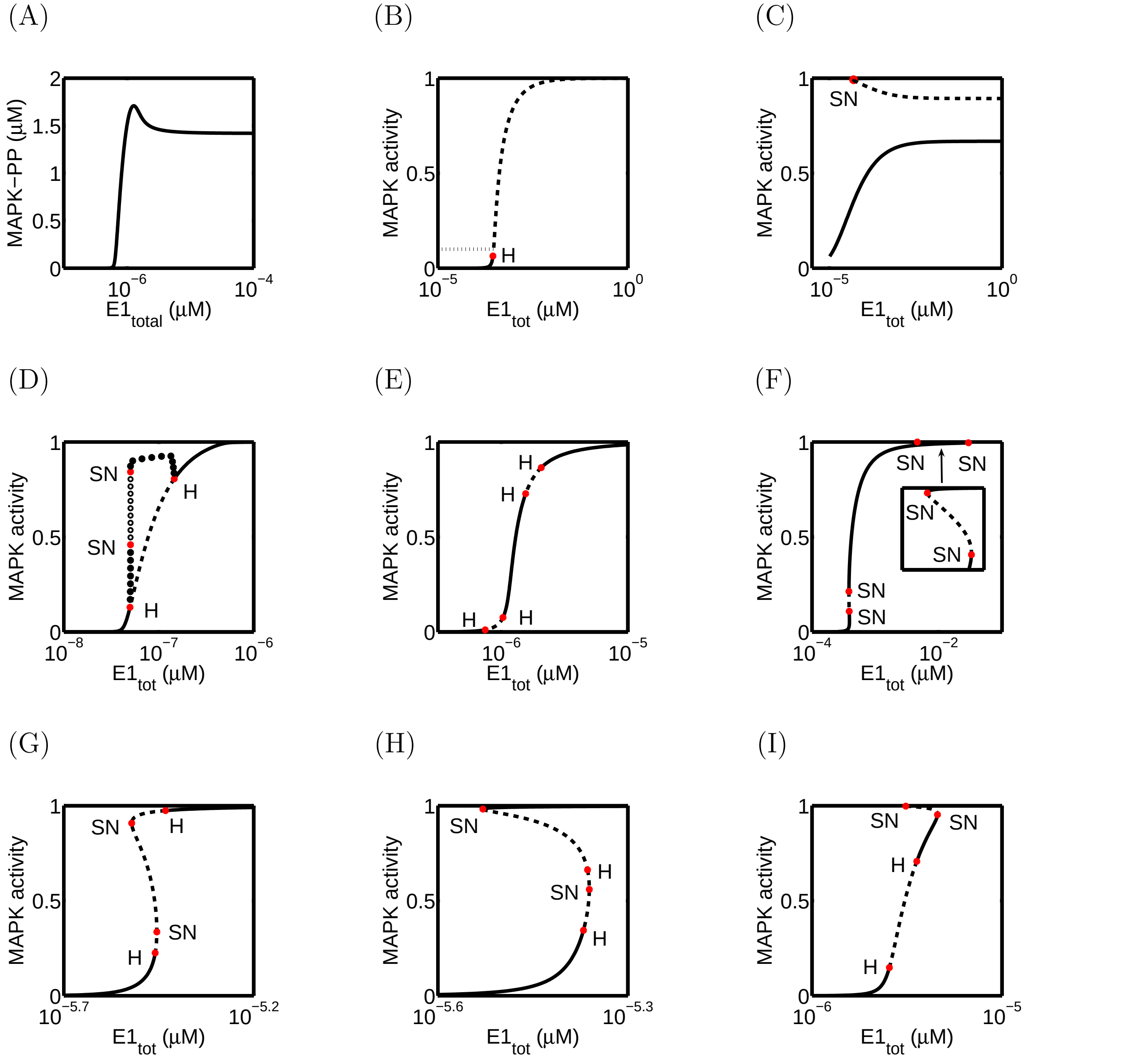

Partial sampling of "complicated" bifurcation diagrams observed in our exploration, along with their automated classification. (A) is classified as "Single-valued". (B), (D) and (E) are classified as "Oscillatory". (C) and (F)-(I) are classified as "Hysteretic".
