# Supplementary material for: Bistability and Oscillations in the Huang-Ferrell Model of MAPK Signaling
Source: PLoS Comput Biol. 2007 Sep 28;3(9):e184. doi: 10.1371/journal.pcbi.0030184 (PMC1994985; doi:10.1371/journal.pcbi.0030184)
Supplement: Figure S3 — (79 KB PDF) [file pcbi.0030184.sg003.pdf]

Figure S3

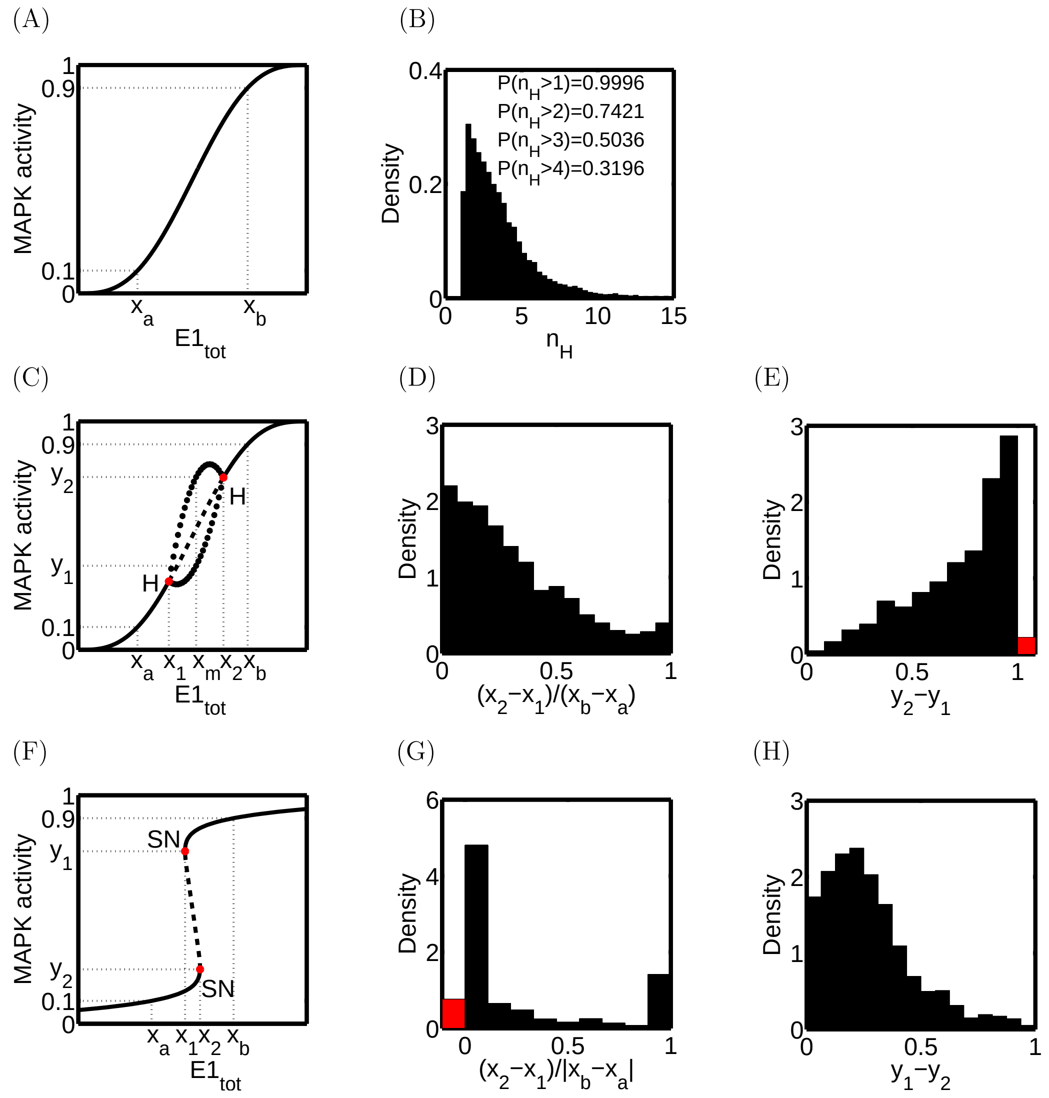

(A), (C) and (F) Schematic plots defining variables useful in the discussion of the adjacent histograms.
(B) Histogram of Hill coefficients ($n_H \equiv \log(81)/\log(x_b/x_a)$) for "Single-valued" bifurcation diagrams; x_a and x_b are inputs ($E1_{tot}$) that correspond to 10% and 90% maximum output (MAPK activity). The quantities x_a and x_b are similarly defined in (C) and (F).
(D) and(E) Histograms for "Oscillatory" bifurcation diagram properties. The quantities x_1 and x_2 in (D) and (E) are defined in (C) as input concentrations at the Hopf bifurcation points. The quantity x_m is defined as $x_m \equiv (x_1 + x_2)/2$. The quantities y_1 and y_2 are the minimum and maximum MAPK activity along the oscillatory solution evaluated at x_m . The red bar in (E) represents the bifurcation diagrams with $y_2 - y_1 > 1$.
(G) and (H) Histograms for "Hysteretic" bifurcation diagrams properties. The quantities x_1 and x_2 in (G) and (H) are defined in (F) as input concentrations at the Saddle-Node bifurcation points. The red bar in (G) represents bifurcation diagrams that are discarded while computing the distributions. The quantities y_1 and y_2 are defined as the MAPK activities at the corresponding Saddle-Node bifurcation points.
