# Supplementary material for: Bistability and Oscillations in the Huang-Ferrell Model of MAPK Signaling
Source: PLoS Comput Biol. 2007 Sep 28;3(9):e184. doi: 10.1371/journal.pcbi.0030184 (PMC1994985; doi:10.1371/journal.pcbi.0030184)
Supplement: Figure S4 — (53 KB PDF) [file pcbi.0030184.sg004.pdf]

Figure S4

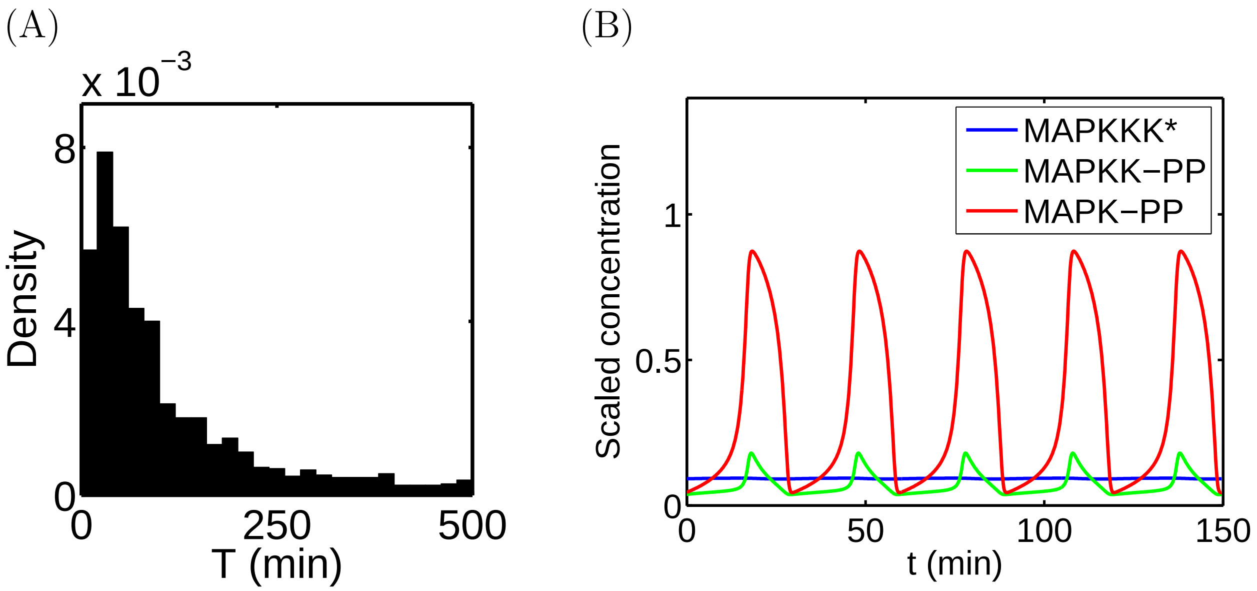

(A) Distribution of the periods of oscillatory solutions for the MAPK cascade. For each "Oscillatory" bifurcation diagram, a single oscillatory solution is evaluated at the point x_m as defined in Figure S3C and the corresponding period is computed. The peak of the distribution is around 30 minutes.

(B) One typical oscillatory solution for the MAPK cascade. The vertical axis corresponds to the concentrations of MAPKKK*, MAPKK-PP and MAPK-PP, scaled by the corresponding total concentrations of MAPKKK, MAPKKK and MAPK respectively.
