# Supplementary material for: Bistability and Oscillations in the Huang-Ferrell Model of MAPK Signaling
Source: PLoS Comput Biol. 2007 Sep 28;3(9):e184. doi: 10.1371/journal.pcbi.0030184 (PMC1994985; doi:10.1371/journal.pcbi.0030184)
Supplement: Table S1 — (51 KB PDF) [file pcbi.0030184.st001.pdf]

Table S1

Parameter	Units	Central value	Range of values
$\text{MAPKKK}_{\text{tot}}$	μM	3×10^{-3}	6×10^{-4} - 1.5×10^{-2}
$\text{MAPKK}_{\text{tot}}$	μM	1.2	0.24 - 6
MAPK_{tot}	μM	1.2	0.24 - 6
E2_{tot}	μM	3×10^{-4}	6×10^{-5} - 1.5×10^{-3}
$\text{MAPKKP}'\text{ase}_{\text{tot}}$	μM	3×10^{-4}	6×10^{-5} - 1.5×10^{-3}
$\text{MAPKP}'\text{ase}_{\text{tot}}$	μM	0.12	2.4×10^{-2} - 0.6
a_i	$(\mu\text{M}\cdot\text{min})^{-1}$	1000	200- 5000
d_i	min^{-1}	150	30- 750
k_i	min^{-1}	150	30- 750
