# Supplementary material for: Bistability and Oscillations in the Huang-Ferrell Model of MAPK Signaling
Source: PLoS Comput Biol. 2007 Sep 28;3(9):e184. doi: 10.1371/journal.pcbi.0030184 (PMC1994985; doi:10.1371/journal.pcbi.0030184)
Supplement: Table S2 — (52 KB PDF) [file pcbi.0030184.st002.pdf]

Table S2

(a)

Parameter	P_1	P_2
a_1	1.0962e+003	3.3723e+002
a_2	1.5576e+003	1.8410e+003
a_3	1.9179e+003	1.2260e+003
a_4	3.6894e+002	2.9603e+003
a_5	4.5836e+003	3.3837e+003
a_6	2.0219e+003	1.9568e+003
a_7	2.6634e+003	2.2957e+002
a_8	1.4435e+003	2.9700e+002
a_9	5.4366e+002	3.3887e+003
a_{10}	4.3990e+002	9.7470e+002
d_1	4.3207e+001	2.6110e+002
d_2	9.4394e+001	1.9847e+002
d_3	7.5216e+001	6.2317e+002
d_4	4.1710e+002	1.6300e+002
d_5	3.3742e+002	6.0531e+002
d_6	4.3905e+002	4.8804e+001
d_7	5.9598e+001	6.9413e+002
d_8	4.0101e+001	3.0109e+002
d_9	2.2211e+002	4.8535e+002
d_{10}	1.7642e+002	5.8745e+002
k_1	6.9525e+001	1.4607e+002
k_2	2.8874e+002	3.3844e+002
k_3	4.3432e+001	4.2000e+002
k_4	7.1505e+002	6.6820e+002
k_5	1.6957e+002	2.1465e+002
k_6	3.4842e+002	6.7970e+001
k_7	4.1954e+001	4.3658e+001
k_8	6.3433e+001	3.1743e+001
k_9	1.1716e+002	6.5732e+001
k_{10}	4.6705e+001	1.7591e+002
$\mathrm{MAPKKK_{tot}}$	3.7112e−003	9.2235e−004
$\mathrm{MAPKK_{tot}}$	4.0658e+000	5.1288e+000
$\mathrm{MAPK_{tot}}$	2.0000e+000	8.1552e−001
$\mathrm{E1_{tot}}$	1.0000e−007	7.9433e−005
$\mathrm{E2_{tot}}$	1.2115e−004	3.2830e−004
$\mathrm{MAPKKP'ase_{tot}}$	9.4579e−005	2.1238e−004
$\mathrm{MAPKP'ase_{tot}}$	7.5372e−002	5.0345e−001

(b)

Concentration	S_1	S_2	S_3	S_4
[KKK· E1]	3.3875e−009	5.4153e−008	5.0486e−008	5.0833e−008
[KKK*]	1.6561e−006	2.0764e−005	1.9358e−005	1.9491e−005
[KKK*·E2]	8.1569e−010	2.3373e−008	2.1790e−008	2.1940e−008
[KK·KKK*]	1.0056e−004	1.4538e−005	1.0146e−004	1.1069e−004
[KK-P]	2.1568e−001	7.3826e−001	4.2017e−001	2.3514e−001
[KK-P·KKP'ase]	6.1078e−006	9.1380e−006	6.3771e−005	6.9576e−005
[KK-P·KKK*]	3.2293e−006	6.3258e−005	3.3564e−005	1.8913e−005
[KK-PP]	7.0439e−003	3.4302e+000	1.4843e−001	4.2903e−002
[KK-PP·KKP'ase]	1.5716e−006	1.9977e−004	1.0600e−004	5.9728e−005
[K·KK-PP]	8.1041e−002	4.0259e−003	2.3989e−002	9.9332e−003
[K-P]	4.9431e−001	1.7081e−002	8.3667e−002	3.1458e−002
[K-P·KP'ase]	5.3600e−002	5.5372e−003	3.2994e−002	1.3662e−002
[K-P·KK-PP]	5.5795e−003	3.6027e−001	7.6363e−002	8.2989e−003
[K-PP]	9.1279e−001	2.9022e−001	5.0569e−002	4.9902e−003
[K-PP·KP'ase]	1.3996e−002	1.3462e−001	2.8535e−002	3.1011e−003
[KKK]	3.6058e−003	8.2371e−004	7.6790e−004	7.7318e−004
[KK]	3.7563e+000	5.9575e−001	4.4595e+000	4.8322e+000
[K]	4.3868e−001	3.7720e−003	5.1941e−001	7.4408e−001
[E1]	9.6612e−008	7.9379e−005	7.9382e−005	7.9382e−005
[E2]	1.2115e−004	3.2828e−004	3.2828e−004	3.2828e−004
[KKP′ase]	8.6900e−005	3.4754e−006	4.2615e−005	8.3078e−005
[KP′ase]	7.7772e−003	3.6329e−001	4.4192e−001	4.8669e−001

(a) Parameter choices leading to an oscillatory solution (P_1) and to multiple steady states (P_2). (b) Steady state solutions for the parameter choices P_1 and P_2. Steady state S_1 is the unstable steady state for P_1. Steady states S_2, and S_4 (for P_2) are stable, while S_3 (also for P_2) is unstable.
