# Supplementary material for: Bistability and Oscillations in the Huang-Ferrell Model of MAPK Signaling
Source: PLoS Comput Biol. 2007 Sep 28;3(9):e184. doi: 10.1371/journal.pcbi.0030184 (PMC1994985; doi:10.1371/journal.pcbi.0030184)
Supplement: Text S1 — (55 KB PDF) [file pcbi.0030184.sd001.pdf]

Text S1

The Huang-Ferrell model is based on the following enzymatic reactions [26]:

$$\mathrm{KKK} + \mathrm{E1} \underset{d_1}{\overset{a_1}{\rightleftharpoons}} \mathrm{KKK} \cdot \mathrm{E1} \xrightarrow{k_1} \mathrm{KKK}^* + \mathrm{E1} \tag{1}$$

$$\mathrm{KKK}^* + \mathrm{E2} \underset{d_2}{\overset{a_2}{\rightleftharpoons}} \mathrm{KKK}^* \cdot \mathrm{E2} \xrightarrow{k_2} \mathrm{KKK} + \mathrm{E2} \tag{2}$$

$$\mathrm{KK} + \mathrm{KKK}^* \underset{d_3}{\overset{a_3}{\rightleftharpoons}} \mathrm{KK} \cdot \mathrm{KKK}^* \xrightarrow{k_3} \mathrm{KK\text{-}P} + \mathrm{KKK}^* \tag{3}$$

$$\mathrm{KK\text{-}P} + \mathrm{KKP'ase} \underset{d_4}{\overset{a_4}{\rightleftharpoons}} \mathrm{KK\text{-}P} \cdot \mathrm{KKP'ase} \xrightarrow{k_4} \mathrm{KK} + \mathrm{KKP'ase} \tag{4}$$

$$\mathrm{KK\text{-}P} + \mathrm{KKK}^* \underset{d_5}{\overset{a_5}{\rightleftharpoons}} \mathrm{KK\text{-}P} \cdot \mathrm{KKK}^* \xrightarrow{k_5} \mathrm{KK\text{-}PP} + \mathrm{KKK}^* \tag{5}$$

$$\mathrm{KK\text{-}PP} + \mathrm{KKP'ase} \underset{d_6}{\overset{a_6}{\rightleftharpoons}} \mathrm{KK\text{-}PP} \cdot \mathrm{KKP'ase} \xrightarrow{k_6} \mathrm{KK\text{-}P} + \mathrm{KKP'ase} \tag{6}$$

$$\mathrm{K} + \mathrm{KK\text{-}PP} \underset{d_7}{\overset{a_7}{\rightleftharpoons}} \mathrm{K} \cdot \mathrm{KK\text{-}PP} \xrightarrow{k_7} \mathrm{K\text{-}P} + \mathrm{KK\text{-}PP} \tag{7}$$

$$\mathrm{K\text{-}P} + \mathrm{KP'ase} \underset{d_8}{\overset{a_8}{\rightleftharpoons}} \mathrm{K\text{-}P} \cdot \mathrm{KP'ase} \xrightarrow{k_8} \mathrm{K} + \mathrm{KP'ase} \tag{8}$$

$$\mathrm{K\text{-}P} + \mathrm{KK\text{-}PP} \underset{d_9}{\overset{a_9}{\rightleftharpoons}} \mathrm{K\text{-}P} \cdot \mathrm{KK\text{-}PP} \xrightarrow{k_9} \mathrm{K\text{-}PP} + \mathrm{KK\text{-}PP} \tag{9}$$

$$\mathrm{K\text{-}PP} + \mathrm{KP'ase} \underset{d_{10}}{\overset{a_{10}}{\rightleftharpoons}} \mathrm{K\text{-}PP} \cdot \mathrm{KP'ase} \xrightarrow{k_{10}} \mathrm{K\text{-}P} + \mathrm{KP'ase} \tag{10}$$

We model the cascade using the following DAE system consisting of 15 ODEs and 7 algebraic equations.

$$\frac{d}{dt}[\mathrm{KKK} \cdot \mathrm{E1}] = a_1[\mathrm{KKK}][\mathrm{E1}] - (d_1 + k_1)[\mathrm{KKK} \cdot \mathrm{E1}] \tag{11}$$

$$\begin{aligned} \frac{d}{dt}[\mathrm{KKK}^*] = & \; k_1[\mathrm{KKK} \cdot \mathrm{E1}] - a_2[\mathrm{KKK}^*][\mathrm{E2}] + d_2[\mathrm{KKK}^* \cdot \mathrm{E2}] \\ & -a_3[\mathrm{KKK}^*][\mathrm{KK}] + (k_3 + d_3)[\mathrm{KK} \cdot \mathrm{KKK}^*] \\ & -a_5[\mathrm{KK\text{-}P}][\mathrm{KKK}^*] + (k_5 + d_5)[\mathrm{KK\text{-}P} \cdot \mathrm{KKK}^*] \end{aligned} \tag{12}$$

$$\frac{d}{dt}[\mathrm{KKK}^* \cdot \mathrm{E2}] = a_2[\mathrm{KKK}^*][\mathrm{E2}] - (d_2 + k_2)[\mathrm{KKK}^* \cdot \mathrm{E2}] \tag{13}$$

$$\frac{d}{dt}[\mathrm{KK} \cdot \mathrm{KKK}^*] = a_3[\mathrm{KK}][\mathrm{KKK}^*] - (d_3 + k_3)[\mathrm{KK} \cdot \mathrm{KKK}^*] \tag{14}$$

$$\begin{aligned}
\frac{d}{dt}[\text{KK-P}] &= k_3[\text{KK}\cdot\text{KKK}^*] - a_4[\text{KK-P}][\text{KKP}'\text{ase}] \\
&\quad + d_4[\text{KK-P}\cdot\text{KKP}'\text{ase}] - a_5[\text{KK-P}][\text{KKK}^*] \\
&\quad + d_5[\text{KK-P}\cdot\text{KKK}^*] + k_6[\text{KK-PP}\cdot\text{KKP}'\text{ase}] \qquad (15)
\end{aligned}$$

$$\frac{d}{dt}[\text{KK-P}\cdot\text{KKP}'\text{ase}] = a_4[\text{KK-P}][\text{KKP}'\text{ase}] - (d_4 + k_4)[\text{KK-P}\cdot\text{KKP}'\text{ase}] \qquad (16)$$

$$\frac{d}{dt}[\text{KK-P}\cdot\text{KKK}^*] = a_5[\text{KK-P}][\text{KKK}^*] - (d_5 + k_5)[\text{KK-P}\cdot\text{KKK}^*] \qquad (17)$$

$$\begin{aligned}
\frac{d}{dt}[\text{KK-PP}] &= k_5[\text{KK-P}\cdot\text{KKK}^*] - a_6[\text{KK-PP}][\text{KKP}'\text{ase}] \\
&\quad + d_6[\text{KK-PP}\cdot\text{KKP}'\text{ase}] - a_7[\text{KK-PP}][\text{K}] \\
&\quad + (d_7 + k_7)[\text{K}\cdot\text{KK-PP}] - a_9[\text{K-P}][\text{KK-PP}] \\
&\quad + (d_9 + k_9)[\text{K-P}\cdot\text{KK-PP}] \qquad (18)
\end{aligned}$$

$$\begin{aligned}
\frac{d}{dt}[\text{KK-PP}\cdot\text{KKP}'\text{ase}] &= a_6[\text{KK-PP}][\text{KKP}'\text{ase}] \\
&\quad - (d_6 + k_6)[\text{KK-PP}\cdot\text{KKP}'\text{ase}] \qquad (19)
\end{aligned}$$

$$\frac{d}{dt}[\text{K}\cdot\text{KK-PP}] = a_7[\text{K}][\text{KK-PP}] - (d_7 + k_7)[\text{K}\cdot\text{KK-PP}] \qquad (20)$$

$$\begin{aligned}
\frac{d}{dt}[\text{K-P}] &= k_7[\text{K}\cdot\text{KK-PP}] - a_8[\text{K-P}][\text{KP}'\text{ase}] \\
&\quad + d_8[\text{K-P}\cdot\text{KP}'\text{ase}] - a_9[\text{K-P}][\text{KK-PP}] \\
&\quad + d_9[\text{K-P}\cdot\text{KK-PP}] + k_{10}[\text{K-PP}\cdot\text{KP}'\text{ase}] \qquad (21)
\end{aligned}$$

$$\frac{d}{dt}[\text{K-P}\cdot\text{KP}'\text{ase}] = a_8[\text{K-P}][\text{KP}'\text{ase}] - (d_8 + k_8)[\text{K-P}\cdot\text{KP}'\text{ase}] \qquad (22)$$

$$\frac{d}{dt}[\text{K-P}\cdot\text{KK-PP}] = a_9[\text{K-P}][\text{KK-PP}] - (d_9 + k_9)[\text{K-P}\cdot\text{KK-PP}] \qquad (23)$$

$$\begin{aligned}
\frac{d}{dt}[\text{K-PP}] &= k_9[\text{K-P}\cdot\text{KK-PP}] - a_{10}[\text{K-PP}][\text{KP}'\text{ase}] \\
&\quad + d_{10}[\text{K-PP}\cdot\text{KP}'\text{ase}] \qquad (24)
\end{aligned}$$

$$\frac{d}{dt}[\text{K-PP}\cdot\text{KP}'\text{ase}] = a_{10}[\text{K-PP}][\text{KP}'\text{ase}] - (d_{10} + k_{10}))[\text{K-PP}\cdot\text{KP}'\text{ase}] \qquad (25)$$

$$\begin{aligned}
0 &= [\text{KKK}] + [\text{KKK}^*] + [\text{KKK}\cdot\text{E1}] + [\text{KKK}^*\cdot\text{E2}] \\
&\quad + [\text{KK}\cdot\text{KKK}^*] + [\text{KK-P}\cdot\text{KKK}^*] - \text{KKK}_{\text{tot}} \qquad (26)
\end{aligned}$$

$$\begin{aligned}
0 &= [\text{KK}] + [\text{KK-P}] + [\text{KK-PP}] + [\text{KK}\cdot\text{KKK}^*] \\
&\quad + [\text{KK-P}\cdot\text{KKK}^*] + [\text{KK-P}\cdot\text{KKP}'\text{ase}] \\
&\quad + [\text{KK-PP}\cdot\text{KKP}'\text{ase}] + [\text{K}\cdot\text{KK-PP}] \\
&\quad + [\text{K-P}\cdot\text{KK-PP}] - \text{KK}_{\text{tot}} \qquad (27)
\end{aligned}$$

$$\begin{aligned}
0 &= [\text{K}] + [\text{K-P}] + [\text{K-PP}] + [\text{K}\cdot\text{KK-PP}] \\
&\quad + [\text{K-P}\cdot\text{KK-PP}] + [\text{K-P}\cdot\text{KP}'\text{ase}] \\
&\quad + [\text{K-PP}\cdot\text{KP}'\text{ase}] - \text{K}_{\text{tot}} \qquad (28)
\end{aligned}$$

$$0 = [\mathrm{E1}] + [\mathrm{KKK} \cdot \mathrm{E1}] - \mathrm{E1}_{\mathrm{tot}} \tag{29}$$

$$0 = [\mathrm{E2}] + [\mathrm{KKK}^* \cdot \mathrm{E2}] - \mathrm{E2}_{\mathrm{tot}} \tag{30}$$

$$0 = [\mathrm{KKP'ase}] + [\mathrm{KK\text{-}P} \cdot \mathrm{KKP'ase}] + [\mathrm{KK\text{-}PP} \cdot \mathrm{KKP'ase}] - \mathrm{KKP'ase}_{\mathrm{tot}} \tag{31}$$

$$0 = [\mathrm{KP'ase}] + [\mathrm{K\text{-}P} \cdot \mathrm{KP'ase}] + [\mathrm{K\text{-}PP} \cdot \mathrm{KP'ase}] - \mathrm{KP'ase}_{\mathrm{tot}} \tag{32}$$
