# Supplementary material for: Bistability and Oscillations in the Huang-Ferrell Model of MAPK Signaling
Source: PLoS Comput Biol. 2007 Sep 28;3(9):e184. doi: 10.1371/journal.pcbi.0030184 (PMC1994985; doi:10.1371/journal.pcbi.0030184)
Supplement: Text S2 — (74 KB PDF) [file pcbi.0030184.sd002.pdf]

Text S2

Equations (26)-(32) as in Text S1 are algebraic constraints due to species conservation laws. They can be rewritten in the following form:

$$
\begin{aligned}
[\mathrm{KKK}] &= \mathrm{KKK_{tot}} - ([\mathrm{KKK}^*] + [\mathrm{KKK}\cdot\mathrm{E1}] + [\mathrm{KKK}^*\cdot\mathrm{E2}] \\
&\quad +[\mathrm{KK}\cdot\mathrm{KKK}^*] + [\mathrm{KK\text{-}P}\cdot\mathrm{KKK}^*]) && (33)\\
[\mathrm{KK}] &= \mathrm{KK_{tot}} - ([\mathrm{KK\text{-}P}] + [\mathrm{KK\text{-}PP}] + [\mathrm{KK}\cdot\mathrm{KKK}^*] \\
&\quad +[\mathrm{KK\text{-}P}\cdot\mathrm{KKK}^*] + [\mathrm{KK\text{-}P}\cdot\mathrm{KKP'ase}] \\
&\quad +[\mathrm{KK\text{-}PP}\cdot\mathrm{KKP'ase}] + [\mathrm{K}\cdot\mathrm{KK\text{-}PP}] \\
&\quad +[\mathrm{K\text{-}P}\cdot\mathrm{KK\text{-}PP}]) && (34)\\
[\mathrm{K}] &= \mathrm{K_{tot}} - ([\mathrm{K\text{-}P}] + [\mathrm{K\text{-}PP}] + [\mathrm{K}\cdot\mathrm{KK\text{-}PP}] \\
&\quad +[\mathrm{K\text{-}P}\cdot\mathrm{KK\text{-}PP}] + [\mathrm{K\text{-}P}\cdot\mathrm{KP'ase}] \\
&\quad +[\mathrm{K\text{-}PP}\cdot\mathrm{KP'ase}]) && (35)\\
[\mathrm{E1}] &= \mathrm{E1_{tot}} - [\mathrm{KKK}\cdot\mathrm{E1}] && (36)\\
[\mathrm{E2}] &= \mathrm{E2_{tot}} - [\mathrm{KKK}^*\cdot\mathrm{E2}] && (37)\\
[\mathrm{KKP'ase}] &= \mathrm{KKP'ase_{tot}} - ([\mathrm{KK\text{-}P}\cdot\mathrm{KKP'ase}] + [\mathrm{KK\text{-}PP}\cdot\mathrm{KKP'ase}]) && (38)\\
[\mathrm{KP'ase}] &= \mathrm{KP'ase_{tot}} - ([\mathrm{K\text{-}P}\cdot\mathrm{KP'ase}] + [\mathrm{K\text{-}PP}\cdot\mathrm{KP'ase}]). && (39)
\end{aligned}
$$

This suggests that we can express Eqns. (11)-(32) as

$$
\begin{aligned}
\dot{\mathbf{x}} &= \mathbf{f}(\mathbf{x}, \mathbf{y}) && (40)\\
0 &= \mathbf{y} + \mathbf{g}(\mathbf{x}), && (41)
\end{aligned}
$$

where $\mathbf{f} : \mathbb{R}^{+22} \rightarrow \mathbb{R}^{15}$, $\mathbf{g} : \mathbb{R}^{+15} \rightarrow \mathbb{R}^{-7}$, $\mathbf{x} \in \mathbb{R}^{+15}$ and $\mathbf{y} \in \mathbb{R}^{+7}$ are the vectors consisting of the concentrations on the left hand side of Eqns. (11)-(25) and Eqns. (33)-(39), respectively. Substituting Eqn. (41) into Eqns. (40) to eliminate $\mathbf{y}$, we reduce the original DAE system (Eqns. (11)-(32)) to a set of 15 ODEs in the following form,

$$\dot{\mathbf{x}} = \mathbf{F}(\mathbf{x}) \tag{42}$$

where $\mathbf{F} : \mathbb{R}^{+15} \rightarrow \mathbb{R}^{15}$ and $\mathbf{F}(\mathbf{x}) \equiv \mathbf{f}(\mathbf{x}, -\mathbf{g}(\mathbf{x}))$.

The (index 1) DAE system (Eqns. (40)-(41)) and the reduced ODE system (Eqn. (42)) clearly share the same steady state solutions. It is easy to see that the stability of the steady state solutions (i.e. the corresponding eigenvalues) is the same.

The eigenvalues of the linearization around a steady state solution $(\mathbf{x}^*, \mathbf{y}^*)$ to Eqns. (40) and (41) are obtained by solving the following generalized eigenproblem:

$$\begin{bmatrix} I_{15\times15} & 0_{15\times7} \\ 0_{7\times15} & 0_{7\times7} \end{bmatrix} \lambda \mathbf{v} = \begin{bmatrix} \mathbf{f}_x & \mathbf{f}_y \\ \mathbf{g}_x & I_{7\times7} \end{bmatrix} \mathbf{v} \tag{43}$$

where the matrices $\mathbf{f}_x \equiv \frac{\partial \mathbf{f}}{\partial \mathbf{x}}$, $\mathbf{f}_y \equiv \frac{\partial \mathbf{f}}{\partial \mathbf{y}}$ and $\mathbf{g}_x \equiv \frac{\partial \mathbf{g}}{\partial \mathbf{x}}$ are evaluated at the steady state, and λ and $\mathbf{v} \in \mathbb{C}^{22}$ denote eigenvalues and the corresponding eigenvectors, respectively. The eigenvector $\mathbf{v}$ can be written as

$$\mathbf{v} = \begin{bmatrix} \mathbf{v}_1 \\ \mathbf{v}_2 \end{bmatrix} \tag{44}$$

where $\mathbf{v}_1 \in \mathbb{C}^{15}$ and $\mathbf{v}_2 \in \mathbb{C}^7$. A trivial eigenvalue $\lambda = \infty$ (with multiplicity 7) is also present. For the nontrivial eigenvalues, substitute Eqn. (44) into Eqn. (43) and we have

$$\lambda \mathbf{v}_1 = \mathbf{f}_x \mathbf{v}_1 + \mathbf{f}_y \mathbf{v}_2 \tag{45}$$

$$0 = \mathbf{g}_x \mathbf{v}_1 + \mathbf{v}_2. \tag{46}$$

Substituting Eqn. (46) into Eqn. (45) to eliminate $\mathbf{v}_2$, we obtain

$$\lambda \mathbf{v}_1 = (\mathbf{f}_x - \mathbf{f}_y \cdot \mathbf{g}_x)\, \mathbf{v}_1. \tag{47}$$

For Eqn. (42), we solve the following eigenvalue problem at the same steady state $\mathbf{x}^*$

$$\tilde{\lambda} \tilde{\mathbf{v}} = \boldsymbol{\mathcal{D}}\mathbf{F} \tilde{\mathbf{v}}, \tag{48}$$

where $\tilde{\mathbf{v}} \in \mathbb{C}^{15}$ and $\boldsymbol{\mathcal{D}}\mathbf{F} \equiv \frac{\partial \mathbf{F}}{\partial \mathbf{x}}|_{\mathbf{x}=\mathbf{x}^*}$ is the Jacobian matrix. Since $\mathbf{F}(\mathbf{x}) \equiv \mathbf{f}(\mathbf{x}, -\mathbf{g}(\mathbf{x})) = \mathbf{f}(\mathbf{x}, \mathbf{y})$ for $\mathbf{y} = -\mathbf{g}(\mathbf{x})$, we have

$$\boldsymbol{\mathcal{D}}\mathbf{F} = \mathbf{f}_x - \mathbf{f}_y \cdot \mathbf{g}_x. \tag{49}$$

Equations (47)-(49) suggest that the nontrivial eigenvalues of Eqns. (40)-(41) are preserved in Eqn. (42) with $\tilde{\mathbf{v}} = \mathbf{v}_1$.
