# Supplementary material for: Bistability and Oscillations in the Huang-Ferrell Model of MAPK Signaling
Source: PLoS Comput Biol. 2007 Sep 28;3(9):e184. doi: 10.1371/journal.pcbi.0030184 (PMC1994985; doi:10.1371/journal.pcbi.0030184)
Supplement: Protocol S1 — (65 KB PDF) [file pcbi.0030184.sd003.pdf]

Protocol S1

We use the following steps to compute the bifurcation diagrams (steady state I/O maps) for randomly sampled parameter sets:

1. Randomly sample the 30 rate constants and 6 total concentrations (except the total concentration of E1 ($E1_{tot}$) which will be used as the distinguished continuation parameter) over a 25-fold range. This is done in $\log_{10}$ space: for each parameter p_i ($i = 1, ..., 36$), the value of $\log_{10} p_i$ is sampled uniformly within [$-\log_{10} 5 + \log_{10} p_{ic}$, $\log_{10} 5 + \log_{10} p_{ic}$]. Each p_{ic} is a predefined constant "central" value as shown in the third column of Table S1.

2. For a given set of sampled 36 parameter values, we fix $E1_{tot} = 1$ μM, and solve for the corresponding steady state concentrations. The initial guess of the steady state solution is obtained by integrating the ODE system for the sampled parameter values and $E1_{tot} = 1$ μM for a period of time ($t = 400$ mins), significantly longer than the *typical* relaxation time of the system. We should note that different initial conditions for this integration may lead to different stable steady states (when they exist). We choose the following initial condition: MAPKKK, MAPKK, and MAPK are initialized in the fully activated form (i.e. MAPKKK*, MAPKK-PP and MAPK-PP respectively); all the other enzymes are not associated with any substrate. When the integration brings the system close to a stable limit cycle solution, the Newton-Raphson for the steady state sometimes fails and in these cases the parameters are saved for further investigation.

3. After a steady state solution for $E1_{tot} = 1$ μM is successfully located, we compute the steady state similarly for $E1_{tot} = 2$ μM (doubling the original "input") and check if the steady state concentration of MAPK-PP (the "output" of the cascade) is changed by less than a small percentage (we choose 0.1%). If not, we perform pseudoarclength continuation from $E1_{tot} = 1$ μM toward larger values of $E1_{tot}$ and repeat the checking process until such a value of $E1_{tot}$, $E1_{tot0}$, is found. Then we perform pseudoarclength continuation from $E1_{tot0}$ towards *lower* values. The sign of the derivative $dE1_{tot}/ds$, where s is the arclength, is monitored using the current and the last two steady states along the branch to detect turning points in $E1_{tot}$.

   We choose the steady state concentration of MAPK-PP at $E1_{tot0}$ to be our base value. We terminate the continuation in the following cases:

   (a) The steady state MAPK-PP concentration at the current step is less than 10% of the base value defined above (for the purpose of computing Hill coefficients).

   (b) The value of $E1_{tot}$ at the current step is larger than 1 μM and $dE1_{tot}/ds > 0$. Meeting this criterion implies that there exists at least one turning point with respect to $E1_{tot}$. Bifurcation diagrams like the one in Figure S2C, which we refer to as "broken" bifurcation diagrams, are the reason for including this criterion. Such diagrams can exhibit (isolated) solution branches extending far to the right of $E1_{tot} = 1$ μM, causing the continuation to break down.

   (c) Exceptions occur, such as physically impossible solutions (i.e. negative concentration values due to numerical inaccuracy) or numerical breakdown (e.g. the maximum number of continuation steps (here 800) is reached or the Newton-Raphson fails to converge after several reductions in the continuation step size down to a minimum prescribed continuation step size). In these cases, the parameters sampled are saved for further investigation.

When the pseudoarclength continuation terminates normally, we *partially* categorize the computed bifurcation diagram through the steps that follow. Exceptions encountered in step 2 and step 3(c) are analyzed individually later to produce the correct bifurcation diagrams for categorization; this may involve some tuning of certain computational parameters (e.g. using different initial conditions, setting larger maximum number of continuation steps and smaller continuation step size).

(a) If at least one turning point in $\mathrm{E1}_{\mathrm{tot}}$ (i.e. the sign changing of $d\mathrm{E1}_{\mathrm{tot}}/ds$) is detected, we categorize it as "Hysteretic". It is important to note that, in our classification, *any* diagram, even one that contains Hopf bifurcations, will be classified as "Hysteretic" as long as a turning point is detected on it.

(b) If no turning point is found in $\mathrm{E1}_{\mathrm{tot}}$ for the computed solution branch, yet at least one bifurcation is detected (i.e. the number of eigenvalues with positive real part changes between two consecutive steady states), we search for Hopf bifurcation points. If at least one of the bifurcations detected is found to be a Hopf bifurcation using the algorithm described below, we categorize the bifurcation diagram as "Oscillatory", otherwise as "Others". Several well-tested algorithms exist for the accurate location of Hopf bifurcation points along a continuation (e.g. the one in [50]). Here, to search for the Hopf bifurcation, we keep track of the eigenvalues for each steady state and monitor the number of eigenvalues with positive real part. When a bifurcation (or possibly bifurcations very close to each other) is detected, we iteratively use bisection to reduce the width of the parameter interval(s) containing at least one bifurcation below a small value (here 10^{-5} in the log10-space). We consider such a small parameter interval to contain a Hopf bifurcation if (1) the difference in the number of eigenvalues with positive real part between the "bounding" steady states is two; and (2) for the bounding steady state possessing larger (resp. smaller) number of eigenvalues with positive real part, we search for those closest and to the right(left) of the imaginary axis. We test for the crossing of an eigenvalue pair by checking that the imaginary parts of these eigenvalues are both nonzero and their difference in magnitude is small (less than 10% of their magnitude average).

(c) If no turning point is found in $\mathrm{E1}_{\mathrm{tot}}$ for the solution branch computed and the number of eigenvalues with positive real part remains unchanged throughout the solution branch computed, the bifurcation diagram is classified as "Single-valued". Otherwise it is characterized as "Oscillatory" (we anticipate Hopf bifurcations outside the parameter range considered in these bifurcation diagrams).

The bifurcation diagrams classified as "Others" in the first computational pass were found, upon closer inspection, to actually be either (a) "Hysteretic", but whose turning points were missed by the sign-changing monitoring subroutine for $d\mathrm{E1}_{\mathrm{tot}}/ds$ due to "large" continuation steps, or (b) "Oscillatory", but with Hopf bifurcation points for which the above conditions were only met across parameter intervals much narrower than 10^{-5} in the log10-space. So for each randomly generated parameter set in our computations, a bifurcation diagram is computed and classified to be one of the three categories ("Hysteretic", "Oscillatory" and "Single-valued"). At the same time, we also check all the bifurcation diagrams computed for local maxima or minima in the steady state concentration of MAPK-PP as a function on $\mathrm{E1}_{\mathrm{tot}}$.

It is important to note that this code contains a number of arbitrary choices and has also several limitations:

(a) Bifurcations on the solution branch for $E1_{tot} > E1_{tot0}$ μM or steady state MAPK-PP concentration lower than 10% of the defined maximum value *are not taken into consideration* (except when just one Saddle-Node bifurcation point has been found, in this case, the continuation is extended until at least one pair of SN points is found).

(b) We choose to classify a bifurcation diagram for which a turning point is found as "Hysteretic" even if it possibly contains additional (even different, e.g. Hopf) bifurcations.

(c) A "broken" bifurcation diagram (like the one in Figure S2C) may be falsely classified as "Single-valued" or "Hopf" instead of "Hysteresis" if the starting point of the continuation happens to fall on the branch without turning points. The systematic initialization described in step 2 (with MAPKKK, MAPKK, and MAPK fully activated and all the other enzymes not associated with any substrate) may help increase the chance of landing on the branch with turning points.

We selected a total of only three classes here (plus "Others") . While the names of our classes ("Single-valued", "Oscillatory" and "Hysteretic") immediately bring to mind "clean" bifurcation diagrams such as those in Figure 2A-2C, many other diagrams are possible, and actually observed. Figure S2 contains a (partial !) sample of the bifurcation diagram variability. The necessity for such a reduced classification is a practical one and contains a certain degree of arbitrariness (we do not, for example, name "Oscillatory" diagrams that contain oscillatory solutions but also contain turning points). We believe that reducing the number of classes (even with some degree of arbitrariness) is necessary in such a statistical study, and urge the reader to keep this in mind.

50. Doedel E, Keller HB, Kernevez JP (1991) Numerical analysis and control of bifurcation problems (I) Bifurcation in finite dimensions. International Journal of Bifurcation and Chaos 1:493-520.
